# Supplementary material for: Archean rifts and triple-junctions revealed by gravity modeling of the southern Superior Craton
Source: Nat Commun. 2025 Oct 6;16:8872. doi: 10.1038/s41467-025-63931-z (PMC12501283; doi:10.1038/s41467-025-63931-z)
Supplement: Supplementary file 1 — Supplementary Information [file 41467_2025_63931_MOESM1_ESM.pdf]

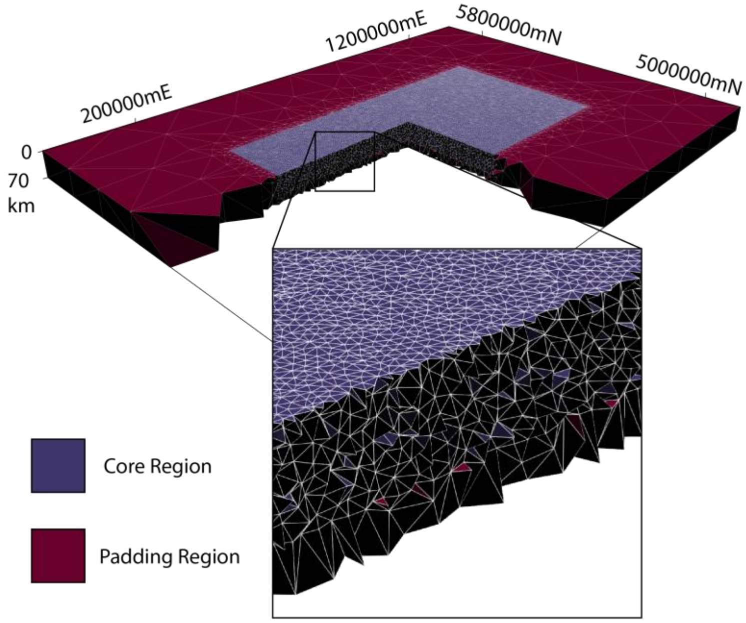

Supplementary Figure 1. An illustration of the mesh used in the modeling of the Abitibi crust and upper mantle. Tetrahedral cells were used to best fit the topography of the region and allow for fine cells in the core of the model (shown in purple) versus the larger cells (shown in red) in the padded region. The padding cells are meant to fit regional trends in the data reflecting density distributions outside of the coverage of the measured gravity data. The mesh in this figure was plotted in the open-source program *ParaView*.

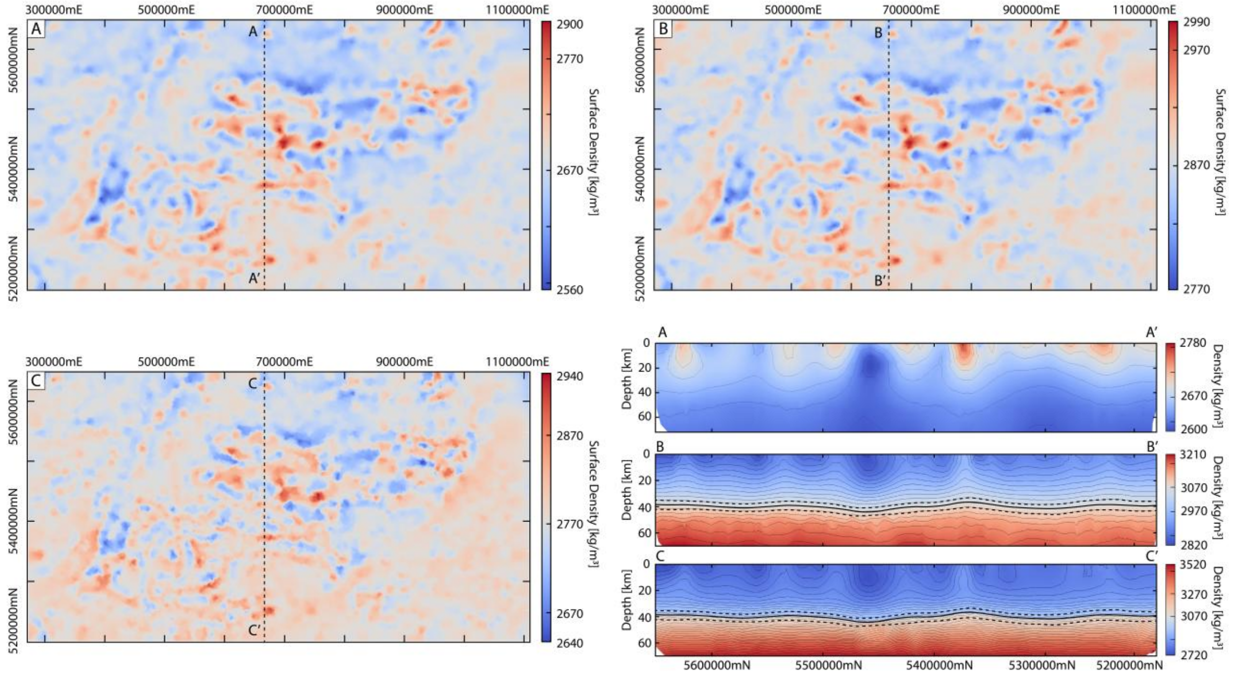

Supplementary Figure 2. Modeling steps of the Abitibi gravity data. A) Map view of the density inversion model produced by inverting the Bouguer data without a refence model. B) Same as A), but inverted with a 1092 mGal constant signal added to the Bouguer data, representing a reference model of relative density 0.0106*z kg/m^3. C) The final model, inverted using the same data as B), but with the Moho surface derived from B) used to constraint the densities in the crust and mantle separately. In cross-sections AA', BB', and CC' the bold black line represents the Moho interface of the model (3070 kg/m^3), the dotted line is the uncertainty envelope of the Moho derived from +/- 20 kg/m^3 contours about the Moho(Galley et al., 2024), and the thin black lines are 20 kg/m^3 contours. The maps are plotted in NAD 1984 UTM Zone 17N.

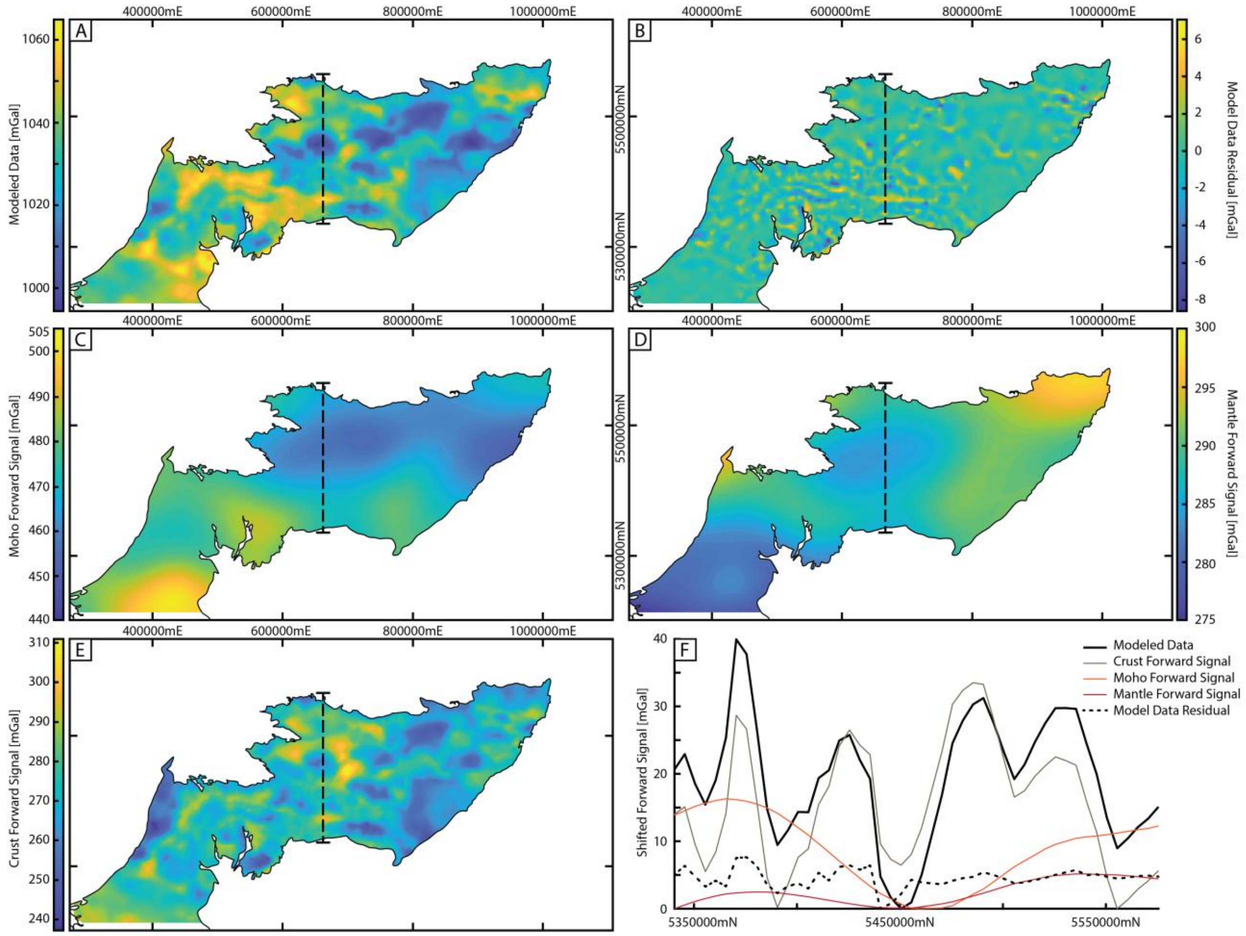

Supplementary Figure 3. The modeled gravity data over the Abitibi. A) the modeled data, including the reference model; B) the model data residual; C) The forward signal from the Moho interface; D) the forward signal from the portion of the model including and below the Moho, i.e., the mantle component; E) the forward signal from the portion of the 3D density model above the Moho, i.e., the crustal component; F) an example section through the data maps (shown as a dotted black line on the maps) showing the relative amplitudes

of the forward signals. The forward signals and data in F) are shifted such that their minimum are at 0 mGal. The maps are plotted in NAD 1984 UTM Zone 17N.
